# Combination of Dabrafenib and Trametinib for the Treatment of Relapsed and Refractory Multiple Myeloma Harboring BRAF V600E Mutation

**DOI:** 10.1155/2020/8894031

**Published:** 2020-10-15

**Authors:** Rūta Čepulytė, Andrius Žučenka, Valdas Pečeliūnas

**Affiliations:** ^1^Vilnius University Hospital Santaros Klinikos, Center of Hematology, Oncology and Transfusion Medicine, Santariškių 2, Vilnius 08661, Lithuania; ^2^Vilnius University Faculty of Medicine, Institute of Clinical Medicine, Clinic of Internal Diseases, Family Medicine and Oncology, Santariškių 2, Vilnius 08661, Lithuania

## Abstract

Multiple myeloma (MM) is an incurable plasma cell neoplasia characterized by relapsed and/or refractory (R/R) disease course, which poses a major therapeutic challenge. New therapies, including BRAF V600E mutation targeting, may become a new treatment option for R/R MM. In combination with mitogen-activated protein kinase inhibitors (MEKi), BRAF inhibitors (BRAFi) could provide better tailored clinical management, although experience in this field is lacking. To this date, there is only one case describing R/R MM treatment with BRAFi vemurafenib and MEKi cobimetinib. This is the first case presenting a R/R MM patient treated with BRAFi dabrafenib and MEKi trametinib.

## 1. Introduction

Multiple myeloma (MM) is an incurable malignancy of mature lymphoid plasma cells located primarily in the bone marrow. Typically, its clinical course is characterized by a pattern of recurrent remissions and relapses, with patients becoming increasingly refractory to treatment [1]. Despite advances in therapy over the last two decades, overcoming drug resistance and salvaging patients with relapsed and/or refractory (R/R) MM are an unmet medical need [2]. Driver mutations in the BRAF gene, a key player in the RAS-RAF-MAPK-ERK pathway, are described in multiple tumor types, including subsets of melanoma, nonsmall cell lung cancer, and anaplastic thyroid cancer, making BRAF a desirable target for inhibition [3]. BRAF V600E, the most common BRAF mutation, has been identified in 2.4% to 5.3% multiple myeloma patients in the Western countries [4–8].

Despite experience in BRAF V600E targeted therapy in melanoma, nonsmall cell lung cancers, and anaplastic thyroid cancer [9], information on efficacy of such MM treatment is very limited. Available data present short-lived responses when treating with BRAF inhibitor (BRAFi) vemurafenib alone [7, 10–12]; thus, inhibition of mitogen‐activated protein kinase (MEK) has emerged as a viable strategy to treat patients with BRAF mutated cancers [2, 13]. The rationale behind adding another RAS-RAF-MAPK-ERK pathway inhibitor to the treatment strategy is based on pathway reactivation [14–18]. When given alone, BRAFi can induce the pathway to become activated evading the blockade. By adding an inhibitor of a downstream effector (MEK), two nodes of the pathway are targeted, thus increasing its suppression [14].

The combination of BRAF and MEK inhibition has shown to improve response rates as well as progression‐free survival (PFS) and overall survival (OS) compared to treatment with BRAF inhibition alone in melanoma [19, 20]. Only very few data are available regarding clinical activity of BRAF and MEK inhibitor combination in R/R MM [21]. To the best of our knowledge, no further case reports of BRAF and MEK kinases inhibitor combination in R/R MM were published. Therefore, we present the first report on BRAF kinase inhibitor dabrafenib and MEK1/MEK2 inhibitor (MEKi) trametinib in the posttransplant and conventional-therapy-resistant BRAF V600E positive MM patient.

## 2. Case Presentation

A 52-year-old male was first diagnosed with immunoglobulin *G* (IgG) kappa light chain (LC) symptomatic MM, Salmon and Durie stage II, in October 2002. Firstly, he presented with pathological fracture due to left femur plasmacytoma and multiple osteolytic lesions. Subsequent examination revealed 21% plasmocytic infiltration in the bone marrow. Risk profiling revealed an International Staging System score of II.

In seven years, he received four lines of MM treatment, alkylators, proteasome inhibitor bortezomib, and two autologous stem cell transplants in May 2009 and February 2016. Progression occurred nine months after the second transplantation, followed by VTD (bortezomib, thalidomide, and dexamethasone) therapy, which was discontinued after one cycle due to refractory disease and infectious complications.

**Figure 1:**
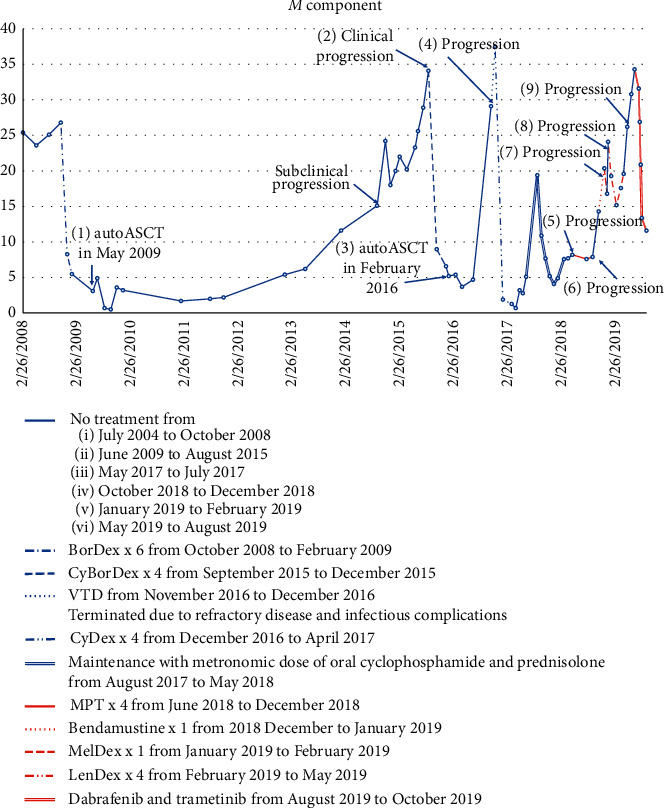
Treatment lines. Data on the *M* component are available from 2009. From 2002 to 2008, the *M* component could not be assessed due to laboratory limitations: (1) consolidation with autologous blood stem cell transplant (autoASCT) in May 2009 included stem cell mobilization and harvesting followed by conditioning with high-dose melphalan (Mel200 mg/m2). (2)In September 2015, the clinical progression manifested with pain in the chest and spine. (3) Consolidation with autologous blood stem cell transplant in February 2016. Conditioning with high-dose melphalan (Mel200 mg/m2), cryopreserved stem cells from 2009 harvest. (4) Nine months after second autoASCT, the fourth clinical progression was diagnosed based on the *M* component rising to 29.1 g/l, kappa LC 1250 mg/l. (5) During maintenance treatment, the new plasmacytoma in lumbar vertebrae occurred. This resulted in left leg paresis and spinal decompression was performed. (6) Worsening of bone pain and *M* component increase. (7) New skull plasmacytoma and *M* gradient increase. (8) New multiple skull plasmacytomas and worsening of bone pain and polyneuropathy, which led to significant decline in functional status. (9) Skull plasmacytomas regressed, but the disease response was minimal due to the stabile *M* component. During the first cycle, the patient experienced proximal left humerus fracture due to a trauma. Osteosynthesis was performed. Two months later, the patient was repeatedly operated due to left upper arm deformation and potential metal construction cutaneous perforation. Biopsy was taken from the left humerus fraction site. During the fourth LenDex cycle, the biopsy revealed plasmocytic infiltration.

Then, the patient received five lines of treatment, including proteasome inhibitor bortezomib, immunomodulators (thalidomide and lenalidomide), bendamustine, and maintenance with alkylators. At that time, daratumumab or carfilzomib was not available. Rapid extramedullary (plasmacytomas of the scull and vertebrae) and systematic progression of the disease followed. The durable response was not achieved in five years (Figure 1).

After 10^th^ line treatment, bone marrow aspiration was performed, and the sample was submitted to genomic profiling in search of possible targeted therapy options. 14q32 (IGH) break-apart was negative, but PCR result of the bone marrow sample was BRAF gene V600E mutation positive. Single nucleotide polymorphism test revealed no clinically significant structural cytogenetic aberrations.

Having informed the patient of the experimental character of a targeted treatment approach in the absence of other available standard options and written informed consent, off‐label use of dabrafenib 150 mg BID in combination with trametinib 2 mg QD was started on 12^th^ August 2019. The treatment was approved by the local ethics committee. Dabrafenib and trametinib were kindly provided by Novartis on compassionate grounds.

Significant reduction of plasmacytomas was observed after just one week of therapy. After 46 days of treatment, *M* component decreased from 30,8 to 13,4 g/l, serum kappa light chain concentration declined from 1720,07 to 577 mg/l, skull plasmacytomas fully regressed. After four lines of sequential therapy, the first partial remission was achieved.

On 27^th^ September, the patient was hospitalized due to febrile fever and diarrhea up to 3 times a day. *Clostridium difficile* colitis was confirmed. During colitis treatment, dabrafenib and trametinib were discontinued due to concerns of possible adverse effect of experimental therapy. After one week of *Clostridium difficile* eradication therapy with metronidazole and negative *Clostridium difficile* PGR test, diarrhea continued with decreasing intensity; therefore, it was considered that BRAFi and MEKi related toxicity. Diarrhea regressed after 3 weeks since discontinuation of the targeted therapy.

**Figure 2:**
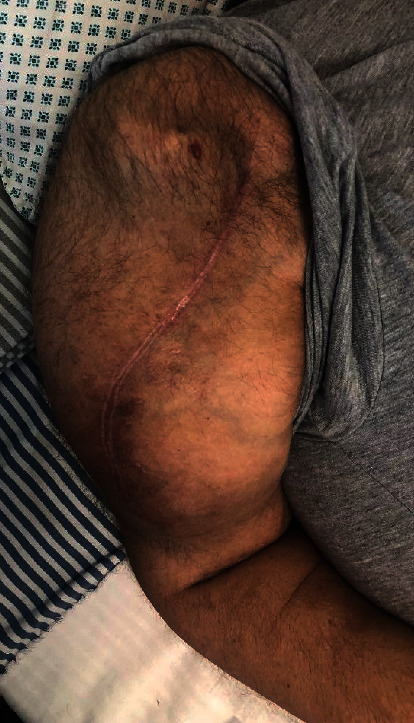
Left upper arm tumor.

**Figure 3:**
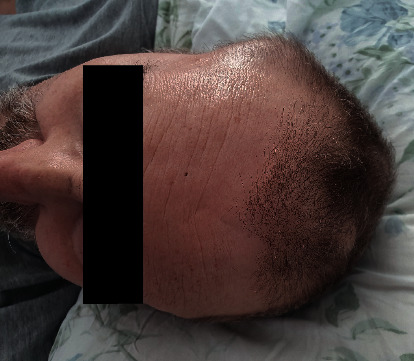
Skull plasmacytomas.

Having discontinued MM treatment for 24 days, new left upper arm 10 × 8 cm tumor (Figure 2) and former skull plasmacytomas (Figure 3) occurred while the *M* component remained stable. BRAFi and MEKi were resumed in reduced dosage to 75 mg BID and 1 mg QD, respectively. After diarrhea regression, dabrafenib and trametinib were continued in standard doses. The patient succumbed to death eleven days after BRAFi and MEKi reinitiation due to MM progression-related complications; therefore, the possibility to regain durable response to this kind of targeted therapy remains unknown.

## 3. Discussion

BRAF mutation is well-known for its activation of the Ras‐Raf‐MEK–ERK signaling pathway, thereby stimulating cellular growth, differentiation, and survival [21, 22]. This mutation has been observed in melanoma, nonsmall cell lung cancer, colorectal carcinoma, and a wide variety of other malignancies, but only some of these diseases have been studied for response to BRAF V600E-specific inhibitors [9, 12].

To date, the published experience in MM is mostly limited to case reports and case series [23]. Majority of them present vemurafenib monotherapy [7, 10–12] or its combination with bortezomib [24]. Upon initiation of vemurafenib, patients rapidly experienced decreased paraprotein levels, extramedullary disease reduction, and improvement of clinical performance status, although duration of response was limited to months since acquired resistance began to develop [11, 12, 24].

The reasons of delayed-onset treatment failure may be associated with spatial genomic heterogeneity in MM [2, 25] and paradoxical activation of the MAPK pathway by BRAF inhibition [26, 27]. Regarding that, a retrospective study conducted in 2016 by Heuck et al. reported 58 R/R MM patients with oncogenic mutations of NRAS, KRAS, or BRAF or GEP pathway activation [13]. All patients received treatment with MEKi trametinib monotherapy with overall response (ORR) of 40%.

The drawbacks of monotherapy encouraged to search for effective treatment combinations. To the best of our knowledge, there is only one case report published that presents combination of BRAFi vemurafenib and MEKi cobimetinib for highly resistant MM patients [21]. After 3 months of treatment, the best response was a very good partial remission (VGPR).

Several prospective studies are now underway and should provide a more comprehensive analysis of efficacy [23]. In the NCI-MATCH study (ClinicalTrials.gov identifier: NCT02465060), R/R MM patients with BRAF V600E/R/K/D mutation in a Phase 2 setting received dabrafenib and trametinib, whereas patients with BRAF fusion or BRAF non-V600 mutation received trametinib and patients with NRAS mutation in codon 12, 13, or 61 received binimetinib. Likewise, dabrafenib in combination with trametinib, dabrafenib alone, or trametinib alone are being evaluated in patients with R/R MM and BRAF/NRAS/KRAS mutations (ClinicalTrials.gov identifier: NCT03091257). In the GMMG-BIRMA Phase 2 study (ClinicalTrials.gov identifier: NCT02834364), R/R MM patients harboring the BRAF V600E/K mutation are being treated with the kinase inhibitor encorafenib (LGX818; RAF kinase inhibitor) in combination with the MEK inhibitor binimetinib (MEK162) [2].

Our report presents a patient with systemic, highly resistant, and rapidly progressing BRAFV600E mutated MM with extramedullary involvement. The disease was refractory to a variety of antimyeloma drugs including melphalan, cyclophosphamide, bortezomib, thalidomide, bendamustine, and lenalidomide. With BRAFV600E and MEK1/2 inhibition approaches, a rapid response was achieved but unsustained due to possible dabrafenib and trametinib toxicity-related discontinuation.

This report illustrates the significance of BRAF/MEK inhibition for patients with R/R MM. However, the duration of response may be limited, and the profile of side effects should be considered in heavily pretreated MM patients. Therefore, it encourages further investigation of therapeutic regimens targeting the BRAF and MEK pathway in patients with R/R MM within clinical trials.
